# Acute and sub-acute oral toxicity of aqueous whole leaf and green rind extracts of *Aloe vera* in Wistar rats

**DOI:** 10.1186/s12906-021-03470-4

**Published:** 2022-01-14

**Authors:** Florence Nalimu, Joseph Oloro, Emanuel L. Peter, Patrick Engeu Ogwang

**Affiliations:** 1 Department of Pharmaceutical Sciences, Faculty of Medicine, Mbarara University of Science and Technology, P.O. Box 1410, Mbarara, Uganda; 2 Department of Pharmacology and Therapeutics, Faculty of Medicine, Mbarara University of Science and Technology, P.O Box 1410, Mbarara, Uganda; 3 Department of Innovation, Technology Transfer & Commercialization, National Institute for Medical Research, Dar es Salaam, Tanzania; 4 Department of Pharmacy, Faculty of Medicine, Mbarara University of Science and Technology, P.O Box 1410, Mbarara, Uganda

**Keywords:** *Aloe vera*, Toxicity, Safety, Aqueous, Whole leaf, Rind

## Abstract

**Background:**

Several local communities in Central, Western, Eastern, and Northern regions of Uganda have been using the whole leaf extracts of *Aloe vera* (L.) Burm. f. (Asphodelaceae) in the treatment of various ailments. Also, several commercial companies sell *A. vera* as soft drinks in Uganda. However, there are inadequate reports on the toxicities of such preparations. This paper reports the acute and sub-acute oral toxicity of aqueous extracts of whole leaf and green rind of *A. vera* in Wistar rats.

**Methods:**

Acute oral toxicity test was carried out in female Wistar rats at doses of 175, 550, 1750, and 5000 mg/kg, p.o. The animals were observed for signs of toxicity for 14 days. Similarly, a sub-acute oral toxicity test was performed in both sexes of rats at doses of 200, 400, and 800 mg/kg, p.o. daily for 28 days. All the groups of animals were monitored for behavioral, morphological, biochemical, and physiological changes, including mortality and compared with respective controls. Body weights were measured weekly while the animals’ relative organ weights, hematological, biochemical, gross, and microscopic pathology were examined on day 29.

**Results:**

There was no mortality or apparent behavioral changes at the doses tested in acute and sub-acute oral toxicity tests. Thus, the Median Lethal Dose (LD_50_) of green rind and whole leaf aqueous extracts was above 5000 mg/kg. Gross anatomy revealed that the rats’ relative spleen weight in green rind extract at 200 mg/kg significantly decreased compared to the control group. The creatinine levels in female rats that received green rind extract and the chloride ion levels in male rats administered whole leaf extract were significantly elevated. Conversely, Mean Corpuscular Hemoglobin Concentration (MCHC) levels significantly decreased at lower doses of the green rind extract compared to the control. Histopathology of the kidney revealed the renal interstitium’s inflammation at doses of 200 and 800 mg/kg of the whole leaf extract.

**Conclusion:**

The findings demonstrated that *A. vera* green rind and whole leaf extracts are non-toxic at relatively high doses when used for a short duration. Prolonged use of the aqueous whole leaf extract might be associated with kidney toxicity.

**Supplementary Information:**

The online version contains supplementary material available at 10.1186/s12906-021-03470-4.

## Background

Herbal medicine plays a significant role in the health of millions of people worldwide through direct utilization and conventional medicine development [1]. Available literature indicates that 60% of the world’s population depends on traditional medicine and 80% of the people in developing countries depend entirely on traditional medicine practices and herbal medicines for their primary health care needs [2]. The continued use of herbal medicine has been viewed to be due to affordability, easy accessibility, lower costs, cultural satisfaction, lack of access to conventional medicine in some areas, and claims of few or no side effects [3, 4]. Also, discontent with conventional medicine, family traditions, and past positive experiences with herbal medicine was among the most commonly cited reasons herbal medicine was opted for [5]. Despite herbal medicines’ consistent use, limited scientific evidence on their safety and efficacy raises concerns among researchers and medicine regulators [6].

*Aloe vera* (L.) Burm. F. (Family: Asphodelaceae) is also known as *Aloe barbadensis*. It is among the most commonly used medicinal plant to manage several ailments among local communities in Uganda [7]. *A. vera* is a stemless or very short-stemmed, evergreen perennial succulent plant growing to 60–100 cm tall, spreading by offsets and root sprouts [8]. *A. vera* leaves contain several phytochemicals such as flavonoids, alkaloids, phenolic acids, tannins, saponin glycosides, anthraquinones, and reducing sugars [9, 10]. In addition, the flowers contain phenolic compounds, flavonoids, and organic acids [11, 12].

The local Ugandan communities use *A. vera* whole leaf decoction for malaria, blood cleansing, stomachache, allergy, yellow fever, worms, fever, urinary tract infections, wasting, and scar removal [13–15]. They also smear leaf extracts on wounds, burns, and skin infections [15]. *A. vera* is also used traditionally in other countries. For example, Indonesians smear the whole leaf juice on skin burns. Also, they use whole leaf gel to treat hair problems [16]. Several studies cited various ethnopharmacological uses of *A. vera* such as antibacterial, antidiabetic, antihypertensive, antihyperlipidemic, antitumor, immunomodulatory, anti-inflammatory, antihypertensive, wound healing, fungicidal, moisturizing, antiviral, and antimutagenic effects [17–19]. Researchers evaluated the toxicity profile of the *A. vera* gel, ethanol whole leaf extract, methanol whole leaf extract, and whole leaf powder [20–24]; however, the toxicity profile of the aqueous extracts of *A. vera* whole leaves has been inadequately reported in Uganda.

Considering the growing local use of decoctions (commonly prepared by boiling the whole leaves in water), particularly as daily soft drinks in Uganda, and the fact that *Aloe vera* whole leaf extract was classified as a possible human carcinogen (Group 2B) by the International Agency for Research on Cancer in 2015 [25], ascertaining the safety of the decoction of the whole leaf and the green rind is paramount to guide the population, the producers, and the regulatory authorities. Often, the rat model is recommended for both acute and subacute toxicity studies due to its reliability in making inferences to human biology [26, 27].

Therefore, this study investigated the acute and sub-acute oral toxicity of the whole leaf and green rind extracts of *A. vera* in Wistar rats.

## Methods

### Chemicals and reagents

These include analytical grades of sulphuric acid, lead acetate, chloroform, acetic acid, and n-butanol (Loba Chemie Pvt. Limited), halothane (Piramal Enterprises Limited, India), HPLC-grade methanol and acetic acid (Sigma-Aldrich, Inc. Germany).

### Plant collection and extraction

Fresh *Aloe vera* leaves were collected from Rwarire at 0°38′55.9″S and 30°38′04.0″E, Mbarara, Uganda, in July 2020 and were authenticated by Mr. Protase Rwaburindori, a plant taxonomist of the Department of Botany, Makerere University. A voucher specimen number FN/011 was deposited in the Makerere University herbarium, Kampala, Uganda.

The leaves were washed under running tap water to remove soil debris and dirt. The spines were removed using a knife. The aqueous whole leaf extract was prepared by first slicing the leaves into small pieces and adding 250 g of the sliced plant material into 500 mL of distilled water. The mixture was extracted by heating at 70 °C for 45 min. The extract was then left to cool, later filtered using a muslin cloth. The aqueous extract was later concentrated under vacuum at 55 °C using a rotary evaporator (IKA, Germany) and lyophilized in a freeze drier (FD-1CL).

The aqueous green rind extract of *A. vera* was prepared by carefully peeling off the outer green epidermis from the leaves and then sliced into small pieces. The extraction procedure was similar to that of the aqueous whole leaf extract. The dried extracts were kept in airtight plastic containers at a temperature of 4 ± 2 °C until needed.

### Phytochemical screening

Preliminary phytochemical screening for alkaloids, tannins, saponins, anthraquinones, amino acids, and flavonoids was analyzed according to standard methods [28].

#### Test for alkaloids (Dragendorff’s test)

To 1 mL of the aqueous extracts in a test tube was added 2 mL of dilute hydrochloric acid followed by 1 mL of Dragendorff’s reagent. An orange-yellow precipitate indicated the presence of alkaloids.

#### Test for tannins (ferric chloride test)

To 1 mL of the aqueous extract in a test tube, 5 drops of 5% Ferric Chloride solution were added. A transient greenish to blue color indicated the presence of tannins.

#### Test for saponins (foam test)

To 5 mL of the aqueous extract in a test tube, 5 mL of distilled water was added, then shaken vigorously and left to stand for 15 min. A persistent froth indicated the presence of saponins.

#### Test for anthraquinones (Borntrager’s test)

About 2 mL of the aqueous extract were boiled with dilute HCl for 2 min, after which the mixture was filtered and cooled. The filtrate was then extracted with chloroform. Then, the chloroform layer was separated and shaken vigorously with 10% ammonium hydroxide. The appearance of rose-pink color in the ammoniacal layer confirmed the presence of anthraquinones.

#### Test for amino acids (Ninhydrin test)

To 2 mL of the aqueous extract in a test tube, 1 mL of Ninhydrin reagent was added and warmed for 1 min. A purple coloration indicated the presence of amino acids.

#### Test for flavonoids (Shinoda’s test)

To 1 mL of the aqueous extract in a test tube, 1 mL of concentrated hydrochloric acid was added, followed by adding a small piece of magnesium. The solution was then boiled for 1 min. The appearance of a reddish pink color indicated the presence of flavonoids.

### HPLC fingerprint

The test sample solutions were prepared by separately dissolving the whole leaf and green rind extracts in distilled water to make a concentration of 1 mg/mL. The samples were then filtered through 0.45 μm membrane filters (EZ-Pak®, France) before loaded to the HPLC system for analysis. The samples were prepared in triplicates. The fingerprints of the whole leaf and green rind extracts of *A. vera* were determined using Reverse HPLC (UFLC Shimadzu, Japan). The chromatographic system comprised a Shimadzu LC-10AT equipped with a communicator CBM-20A (Tokyo, Japan), degassing unit DGU-20A_5R_ (USA), an LC-20 AD pump coupled with an SPD-20A UV/VIS detector (Tokyo, Japan). The HPLC separation was performed on a Luna® C18 column (5 μm; 250 × 4.6 mm; Phenomenex, U.S.A.) maintained at 25 °C in a Shimadzu column oven (CTO-20 AC, Tokyo Japan). It proceeded via isocratic elution with a mobile phase system methanol: 1% acetic acid in water (3:7 v/v) with an injection volume of 20 μL at a flow rate of 0.6 mL/min and detection at a wavelength of 254 nm.

### Experimental animals

Healthy male and female Wistar albino rats (*Rattus norvegicus*), aged 8–12 weeks (100–250 g) were obtained from Mbarara University of Science and Technology (MUST). The animals (each sex differently) were kept in plastic cages and maintained at a temperature of 28 ± 2 °C, and relative humidity ranged 45–55% under a 12 h natural dark/light cycle. The rats were acclimatized for 7 days with free access to standard pellets and clean drinking water ad libitum.

The animals were individually labeled with permanent markers for easy identification. The animal handling followed the guidelines for the care and use of laboratory animals [29], and an institutional ethical review (02/02–20) was obtained before carrying out the study. For each extract’s acute toxicity study, 3 females were used for each dose level, while for the sub-acute toxicity study, 7 groups were used, each comprising 6 animals, 3 males, and 3 females. Lamorte’s power analysis calculator was adopted for sample size estimation in each group for the sub-acute toxicity study. The desired power of 90% at a *p*-value of 0.05 gave the sample size of 5 animals per group. According to OECD guideline 407 [27], each group should have 10 animals (5 males and 5 females). However, following the 3R principles in animal research implementation, 6 animals (3 males and 3 females) were used in each group. Therefore, 66 animals were used for the animal experiment. During the study, the rats of the same sex belonging to the same group were caged together. The Animal Research: Reporting of In Vivo Experiments (ARRIVE) guidelines [30] were used for reporting the study results, and the ARRIVE checklist is included as an Additional file 1.

### Acute Oral toxicity study

The acute oral toxicity study on the whole leaf and green rind extracts of *A. vera* was conducted according to the ‘Up and Down’ method described in OECD guideline 425 [26]. Nulliparous, non-pregnant female Wistar albino rats were used. For each extract, the animals were randomly divided into 4 groups, each having 3 rats. A single animal was administered a starting dose of 175 mg/kg and observed for a period of 48 h [26]. The animal was observed continuously for the first 30 min post-administration, every 1 h for 4 h, then once daily for 48 h for signs of toxicity including bodyweight variation, salivation, tremors, convulsions, diarrhea, changes in the skin, fur, eyes, and mucous membranes, or death before the next doses of 550, 1750 and 5000 mg/kg were administered. In addition, the animals were monitored daily for additional 12 days for delayed signs of toxicity and mortality. The body weights were taken on day 0, day 7, and day 14. Finally, the LD50 was determined.

### Sub-acute oral toxicity Test

A sub-acute oral toxicity study was carried out using 28 days Repeated Dose Toxicity Method as described in OECD guideline 407 [27]. Adult Wistar albino rats of both sexes were divided into seven (7) groups of six (6) animals each; 3 male and 3 females). Group I (control group) received 10 mL/kg of distilled water; groups II-1 V received 200, 400 and 800 mg/kg of *A. vera* whole leaf extract, while groups V-VII received 200, 400 and 800 mg/kg of *A. vera* green rind extract. The treatments were given by oral gavage once daily for 28 days. In addition, the animal body weights were taken weekly, including observations for any physiological or behavioral changes as in the acute toxicity test.

On day 29, the animals were fasted overnight with free access to water, and then anesthetized by exposing them to 4% halothane vapor in a closed chamber. Blood was collected from each animal via cardiac puncture into EDTA coated tubes and non-EDTA tubes for hematological and biochemical analyses, respectively, after which the animals were sacrificed by cervical dislocation. Later, the organs (brain, liver, kidneys, spleen, ovary, and testes) were carefully removed from the dissected rats, washed with distilled water, weighed, and examined macroscopically for any gross changes. The liver and the kidneys were then fixed in 10% neutral buffered formalin in labeled bottles for preservation and preparation for histopathological examination. Each harvested organ’s relative weight was also calculated from formula 1 below [31].

1 $$\mathrm{Relative}\ \mathrm{organ}\ \mathrm{weight}\ \left(\%\right)=\frac{Organ\ weight}{Body\ weight}\times 100$$

### Assessment of hematological parameters

The hematological analysis was performed on blood samples collected in EDTA-coated tubes using a Mindray hematology analyzer. The hematological parameters analyzed included total and differential white blood cell (WBC) count, Red Blood Cells (RBC), Red Cell Distribution Width (RCDW), hemoglobin (Hb), hematocrit (HCT), mean corpuscular volume (MCV), mean corpuscular hemoglobin (MCH), mean corpuscular hemoglobin concentration (MCHC), platelet count (PLT) and mean platelet volume (MPV).

### Assessment of serum biochemical parameters

Blood samples in non-EDTA coated tubes were allowed to clot for 5 min and immediately centrifuged at 3000 rpm for 10 min to separate serum for analysis. Aspartate aminotransferase (AST), Alanine aminotransferase (ALT), urea, creatinine, and total cholesterol were analyzed using a chemistry analyzer (Humastar 200, Germany), while the electrolytes were analyzed using an electrolyte analyzer (Humlyte plus 5, Germany).

### Histopathological analysis

Representative sections of the liver and the kidney were made and fixed in tissue cassettes and processed in a tissue processor for twelve hours. The cassettes were embedded in molten paraffin wax and left to form blocks on a cold plate. The paraffin blocks were mounted on a rotary microtome to make sections with 4 μm thick cells. These sections were then carefully removed from the microtome knife and put in a water bath to remove folds. The best sections were mounted on labeled slides and placed in an oven at 58 °C overnight to fix. The slides were removed and stained using the Harris hematoxylin & eosin technique. A mountant (Dibutyl phthalate in xylene) was added thereafter, and finally covered with a cover slip to prevent the formation of bubbles. The slides were examined under a standard light microscope (× 200 and × 400) for any lesions or abnormalities.

### Statistical analysis

The computer-guided Statistical Programme-AOT425StatPgm (version 1.0) and GraphPad Prism software Version 8.0.2 were used for analysis. The analysis involved summarized data into mean ± SEM using descriptive statistics. Next, the Shapiro-Wilk test was performed to test for normality, after which One-way ANOVA or the Kruskal-Wallis test was performed. This was followed by Tukey’s post hoc test for multiple comparisons. Differences in the mean between the treatment groups were considered significant at *p* < 0.05.

## Results

### Phytochemical screening results

Phytochemical investigations showed the presence of tannins, anthraquinones, and amino acids as major phytochemical groups in addition to alkaloids, saponins, and flavonoids in the whole leaf and green extracts of *A. vera* (Table 1).

**Table 1:** Phytochemical groups present in *A. vera*

Phytochemicals	*A. vera* green rind extract	*A. vera* whole leaf extract
Alkaloids	+	+
Saponins	+	+
Tannins	+++	+++
Flavonoids	++	++
Anthraquinones	+++	+
Amino acids	+++	+++

+ traces present; ++ moderate present; +++ adequate amount

### HPLC fingerprints of the whole leaf and green rind of *A. vera*

**Fig. 1:**
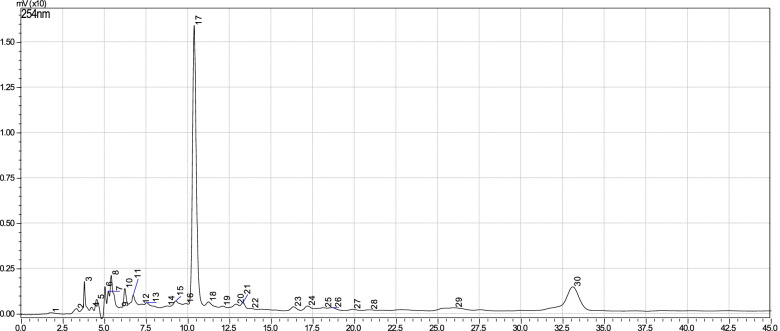
HPLC fingerprint of the whole leaf of *A. vera*

**Fig. 2:**
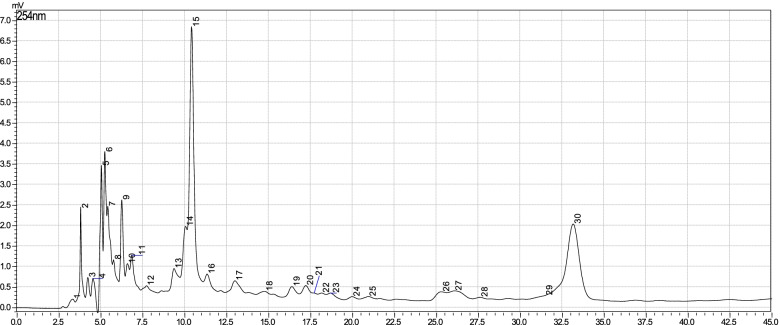
HPLC fingerprint of the green rind of *A. vera*

The HPLC chromatograms of the aqueous whole leaf (Fig. 1) and green rind extracts (Fig. 2) of *A. vera* showed 30 chromatographic peaks each. Peak 17 of the whole leaf extract and peak 15 of the green rind extract exhibited the same retention time (*Rt*) of 10.5 min. Peak 30 for both extracts showed the same *Rt* of 32.0 min.

### Acute oral toxicity effects of aqueous whole leaf and green rind extracts of *A. vera*

**Fig. 3:**
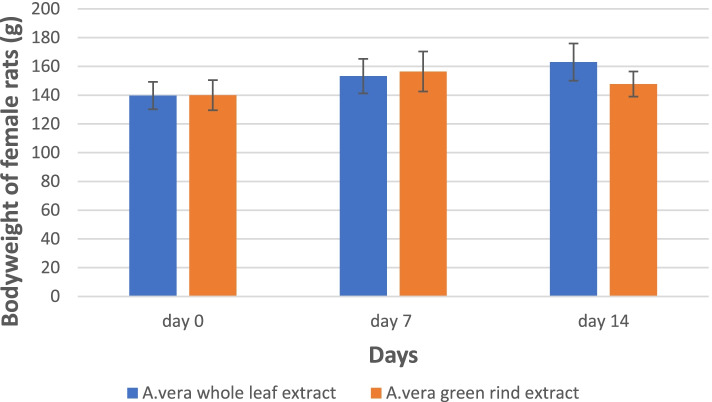
Body weight of female rats after a single exposure to the whole leaf and green rind extracts

All the extracts caused no mortality and behavioral changes in the rats throughout the period of study. Generally, there was an increase in the animals’ bodyweight throughout the study period (Fig. 3). Therefore, the LD_50_ was estimated to be above 5000 mg/kg for all the extracts.

### Sub-acute oral toxicity effects of aqueous whole leaf and green rind extracts of *A. vera*

#### Physical and behavioral signs of toxicity

There was no mortality in all the groups except for one female rat in the control group that died on day 25, whose histopathology examination revealed tubular atrophy and inflammation of the renal interstitium. This animal was excluded from the analysis. There were no apparent behavioral changes in all the treated animals.

#### Effect of aqueous whole leaf and green rind extracts of *A. vera* on the bodyweight of the rats

**Fig. 4:**
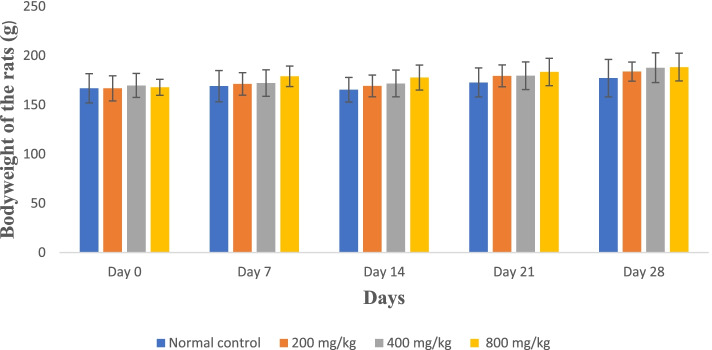
Mean body weight of rats treated with the whole leaf extract for 28 days

**Fig. 5:**
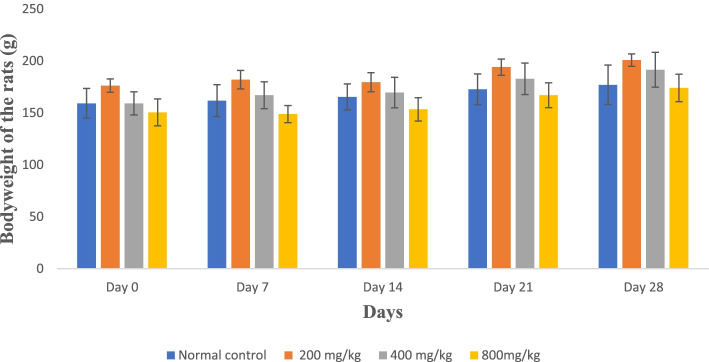
Mean body weight of rats treated with the green rind extract for 28 days

There were no significant variations (*p* > 0.05) in all the treated rats’ mean body weights at all doses compared to the control group throughout the 28 days (Fig. 4 and Fig. 5).

### The relative organ weight

Generally, there were no significant (*p* > 0.05) variations in the relative weights of the liver, kidney, brain, ovary, and testes of the rats among the different groups compared with the control (Table 2). However, the relative spleen weight significantly decreased in rats treated with 200 mg/kg of *A. vera* green rind extract. Moreover, there was no significant difference in mean relative organ weights between male and female rats (Table 2).

**Table 2:** Relative organ weight of the rats treated with *Aloe vera* for 28 days

Organ	Control group	***A. vera*** whole leaf extract doses in mg/kg			***A. vera*** green rind extract doses in mg/kg		
		200	400	800	200	400	800
Combined male and female							
Liver	4.70 ± 0.13	4.50 ± 0.18	4.36 ± 0.17	4.48 ± 0.07	4.49 ± 0.13	4.50 ± 0.21	4.79 ± 0.22
Kidney	0.91 ± 0.04	0.88 ± 0.03	0.87 ± 0.03	0.90 ± 0.02	0.83 ± 0.02	0.84 ± 0.02	0.87 ± 0.03
Brain	0.98 ± 0.08	0.87 ± 0.07	0.93 ± 0.07	0.95 ± 0.06	0.85 ± 0.08	0.89 ± 0.07	1.07 ± 0.14
Spleen	0.59 ± 0.03	0.50 ± 0.05	0.56 ± 0.04	0.47 ± 0.04	0.41 ± 0.05**	0.47 ± 0.03	0.45 ± 0.03
Females							
Liver	4.52 ± 0.04	4.77 ± 0.11	4.61 ± 0.11	4.52 ± 0.10	4.70 ± 0.03	4.72 ± 0.36	5.11 ± 0.16
Kidney	0.94 ± 0.07	0.89 ± 0.06	0.89 ± 0.08	0.91 ± 0.02	0.85 ± 0.00	0.86 ± 0.03	0.91 ± 0.06
Brain	1.12 ± 0.02	0.98 ± 0.03	0.99 ± 0.13	0.96 ± 0.01	0.95 ± 0.05	0.99 ± 0.09	1.30 ± 0.15
Spleen	0.60 ± 0.07	0.49 ± 0.02	0.55 ± 0.08	0.42 ± 0.04	0.45 ± 0.03	0.51 ± 0.01	0.40 ± 0.04
Ovary	2.21 ± 0.44	2.63 ± 0.06	3.21 ± 0.46	1.83 ± 0.35	2.15 ± 0.54	2.01 ± 0.43	2.05 ± 0.15
Males							
**Liver**	4.88 ± 0.18	4.24 ± 0.20	4.12 ± 0.20	4.44 ± 0.15	4.28 ± 0.09	4.28 ± 0.19	4.46 ± 0.23
**Kidney**	0.89 ± 0.05	0.87 ± 0.06	0.86 ± 0.04	0.90 ± 0.05	0.80 ± 0.04	0.82 ± 0.02	0.84 ± 0.01
**Brain**	0.85 ± 0.05	0.76 ± 0.07	0.87 ± 0.07	0.94 ± 0.14	0.75 ± 0.13	0.78 ± 0.03	0.85 ± 0.00
**Spleen**	0.58 ± 0.04	0.51 ± 0.13	0.58 ± 0.04	0.51 ± 0.05	0.37 ± 0.11	0.43 ± 0.03	0.49 ± 0.03
**Testis**	2.76 ± 0.32	2.53 ± 0.22	2.81 ± 0.35	2.94 ± 0.33	2.69 ± 0.44	2.87 ± 0.23	2.75 ± 0.17

Data are expressed as Mean ± SEM; number of rats per group, *n* = 4 for analysis by group and *n* = 3 for separate sexes analysis **significant *p*-value when control is compared with male and female combined

### Hematological indices

Except for eosinophils, Hematocrit (HCT), Mean Corpuscular Hemoglobin Concentration (MCHC), and Mean Platelet Volume (MPV), there were no statistically significant (*p* > 0.05) variations in the rats’ hematological parameters among the different groups (Table 3). However, the mean values of HCT, Mean Corpuscular Hemoglobin (MCH), and MPV significantly increased while MCHC of the females and male rats significantly decreased compared to their respective controls (Table 3). The mean Hemoglobin (Hb) levels significantly increased in females treated with 200 mg/kg of *A. vera* whole leaf extract. Similarly, the mean HCT significantly increased in females treated with 200 and 400 mg/kg of *A. vera* whole leaf extract. Furthermore, a significant increase was observed in mean MPV values in female rats treated with 200 mg/kg of *A. vera* green rind and all doses of *A. vera* whole leaf extract. Significantly lower levels of MCH were recorded in males treated with 800 mg/kg of *A. vera* whole leaf extract and 400 mg/kg of *A. vera* green rind extract.

**Table 3:** Hematological profile of rats treated with *A. vera* for 28 days

Hematological parameters	Control group	***A. vera*** whole leaf extract dose levels in mg/kg			***A. vera*** green rind extract dose levels in mg/kg		
		200	400	800	200	400	800
Combined male and female							
WBC (× 10^9^/L)	11.8 ± 1.24	11.6 ± 1.18	13.1 ± 2.60	13.1 ± 0.57	9.9 ± 1.06	9.9 ± 1.53	12.0 ± 1.6
NEU (× 10^9^/L)	2.3 ± 0.40	3.0 ± 0.54	3.2 ± 0.79	2.3 ± 0.19	2.3 ± 0.29	2.4 ± 0.46	2.9 ± 0.33
LYM(× 10^9^/L)	8.3 ± 1.14	7.2 ± 0.89	8.4 ± 1.89	9.4 ± 0.50	6.4 ± 1.02	6.4 ± 0.92	7.7 ± 1.45
MONO (× 10^9^/L)	0.8 ± 0.17	0.7 ± 0.09	1.0 ± 0.21	0.8 ± 0.11	0.7 ± 0.08	0.6 ± 0.17	0.8 ± 0.14
EOS (× 10^9^/L)	0.27 ± 0.06	0.64 ± 0.09**	0.43 ± 0.09	0.49 ± 0.08	0.4 ± 0.06	0.4 ± 0.15	0.5 ± 0.06
BASO (× 10^9^/L)	0.002 ± 0.002	0.0 ± 0.00	0.0 ± 0.00	0.0 ± 0.00	0.0 ± 0.00	0.0 ± 0.00	0.0 ± 0.00
RBC (× 10^12^/L)	7.7 ± 0.50	8.4 ± 0.35	8.6 ± 0.16	8.8 ± 0.37	8.6 ± 0.23	8.7 ± 0.37	8.7 ± 0.23
Hb (g/dL)	13.7 ± 0.81	14.5 ± 0.68	15.0 ± 0.21	15.4 ± 0.37	15.3 ± 0.38	15.2 ± 0.56	15.6 ± 0.34
HCT (%)	41.0 ± 2.71	46.0 ± 2.25	46.2 ± 0.81	47.62 ± 1.03	48.97 ± 0.80**	48.70 ± 1.56**	48.33 ± 1.30**
MCV (fL)	53.12 ± 1.23	54.73 ± 1.15	53.73 ± 1.03	54.12 ± 1.43	56.87 ± 0.72	56.10 ± 1.62	55.12 ± 0.40
MCH (pg)	17.82 ± 0.26	17.38 ± 0.23	17.55 ± 0.18	17.53 ± 0.33	17.78 ± 0.09	17.50 ± 0.35	17.88 ± 0.11
MCHC (g/dL)	33.58 ± 0.42	31.73 ± 0.33**	32.68 ± 0.30	32.45 ± 0.37	31.30 ± 0.37**	31.30 ± 0.40**	32.47 ± 0.21
RDW-CV (%)	19.22 ± 1.33	19.68 ± 0.58	20.22 ± 0.35	20.35 ± 0.72	19.28 ± 0.25	19.48 ± 0.83	19.93 ± 0.87
PLT(× 10^9^/L)	886.80 ± 143.80	732.0 ± 24.7	766.0 ± 49.0	742.7 ± 24.7	743.7 ± 46.53	774.0 ± 26.41	807.3 ± 68.63
Females							
WBC (× 10^9^/L)	10.72 ± 3.11	12.56 ± 1.02	14.28 ± 5.23	12.63 ± 1.05	10.98 ± 1.87	8.42 ± 1.23	10.48 ± 0.77
NEU (× 10^9^/L)	1.74 ± 0.14	2.32 ± 0.13	2.90 ± 0.77	2.36 ± 0.29	2.19 ± 0.16	1.87 ± 0.45	2.36 ± 0.13
LYM (× 10^9^/L)	9.32 ± 1.77	8.73 ± 0.72	9.63 ± 4.00	9.11 ± 1.05	7.58 ± 1.74	5.79 ± 0.66	6.77 ± 0.70
MONO (× 10^9^/L)	0.83 ± 0.12	0.78 ± 0.06	1.27 ± 0.37	0.78 ± 0.23	0.77 ± 0.09	0.45 ± 0.11	0.90 ± 0.14
EOS (× 10^9^/L)	0.19 ± 0.06	0.73 ± 0.18	0.32 ± 0.06	0.37 ± 0.05	0.45 ± 0.09	0.31 ± 0.08	0.46 ± 0.09
BASO (× 10^9^/L)	0.00 ± 0.00	0.00 ± 0.00	0.00 ± 0.00	0.00 ± 0.00	0.00 ± 0.00	0.00 ± 0.00	0.00 ± 0.00
RBC (× 10^12^/L)	7.85 ± 0.39	8.86 ± 0.26	8.39 ± 0.13	8.06 ± 0.19	8.49 ± 0.37	8.11 ± 0.22	8.49 ± 0.31
Hb (g/dL)	13.95 ± 0.05	15.77 ± 0.49*	15.03 ± 0.19	14.67 ± 0.18	15.07 ± 0.64	14.67 ± 0.60	15.37 ± 0.52
HCT (%)	41.65 ± 0.65	49.23 ± 1.13*	46.87 ± 0.99*	45.70 ± 0.64	48.87 ± 1.07	47.90 ± 1.74	47.23 ± 2.14
MCV (fL)	53.25 ± 3.45	56.80 ± 1.24	55.87 ± 0.84	56.80 ± 1.20	57.67 ± 1.26	59.07 ± 1.32	55.57 ± 0.58
MCH (pg)	17.80 ± 0.80	17.83 ± 0.15	17.93 ± 0.15	18.20 ± 0.25	17.77 ± 0.17	18.07 ± 0.47	18.10 ± 0.10
MCHC (g/dL)	33.50 ± 0.60	31.37 ± 0.58	32.10 ± 0.32	32.10 ± 0.55	30.83 ± 0.65*	30.60 ± 0.17*	32.53 ± 0.37
RDW-CV (%)	16.85 ± 2.05	18.60 ± 0.40	19.83 ± 0.32	19.30 ± 0.99	19.27 ± 0.53	17.97 ± 0.58	18.37 ± 0.93
PLT(× 10^9^/L)	706.0 ± 14.29	722.0 ± 23.18	808.3 ± 61.87	734.0 ± 26.63	799.7 ± 52.14	793.7 ± 45.95	855.3 ± 139.0
MPV (fL)	7.35 ± 0.15	8.27 ± 0.09*	8.23 ± 0.09*	8.20 ± 0.20*	9.33 ± 0.19*	8.60 ± 0.40	8.23 ± 0.35
Males							
WBC (× 10^9^/L)	12.54 ± 1.69	10.68 ± 2.24	12.08 ± 2.34	13.74 ± 0.46	8.99 ± 1.07	11.45 ± 2.82	13.56 ± 3.24
NEU (× 10^9^/L)	2.77 ± 0.56	3.69 ± 0.98	3.58 ± 1.56	2.32 ± 0.32	2.49 ± 0.61	2.94 ± 0.75	3.55 ± 0.43
LYM(× 10^9^/L)	8.61 ± 1.70	5.72 ± 1.09	7.20 ± 0.57	9.69 ± 0.28	5.38 ± 0.97	7.03 ± 1.85	8.65 ± 3.01
MONO (× 10^9^/L)	0.92 ± 0.29	0.71 ± 0.19	0.92 ± 0.23	0.91 ± 0.05	0.70 ± 0.15	0.89 ± 0.30	0.83 ± 0.29
EOS (× 10^9^/L)	0.35 ± 0.03	0.56 ± 0.04	0.38 ± 0.08	0.67 ± 0.05	0.42 ± 0.10	0.58 ± 0.29	0.53 ± 0.09
BASO (× 10^9^/L)	0.0033 ± 0.0033	0.00 ± 0.00	0.00 ± 0.00	0.00 ± 0.00	0.00 ± 0.00	0.00 ± 0.00	0.00 ± 0.00
RBC (× 10^12^/L)	7.66 ± 0.88	7.94 ± 0.59	8.80 ± 0.26	9.64 ± 0.09	8.76 ± 0.33	9.32 ± 0.54	9.04 ± 0.29
Hb (g/dL)	13.63 ± 1.47	13.40 ± 0.82	15.10 ± 0.44	16.23 ± 0.17	15.60 ± 0.49	15.83 ± 0.94	16.00 ± 0.46
HCT (%)	40.67 ± 4.91	41.73 ± 2.44	45.43 ± 1.34	49.53 ± 1.09	49.07 ± 1.43	49.50 ± 2.91	49.43 ± 1.62
MCV (fL)	53.03 ± 1.02	52.67 ± 0.90	51.60 ± 0.15	51.43 ± 1.28	56.07 ± 0.58	53.13 ± 1.59	54.67 ± 0.50
MCH (pg)	17.83 ± 0.15	16.93 ± 0.22*	17.17 ± 0.03	16.87 ± 0.17*	17.80 ± 0.12	16.93 ± 0.23*	17.67 ± 0.07
MCHC (g/dL)	33.63 ± 0.68	32.10 ± 0.26	33.27 ± 0.03	32.80 ± 0.50	31.77 ± 0.18	32.00 ± 0.52	32.40 ± 0.26
RDW-CV (%)	20.80 ± 1.18	20.77 ± 0.57	20.60 ± 0.61	21.40 ± 0.72	19.30 ± 0.17	21.00 ± 0.91	21.50 ± 0.66
PLT(× 10^9^/L)	1014 ± 220.6	742.0 ± 49.14	723.7 ± 80.05	751.3 ± 47.64	687.7 ± 70.48	754.3 ± 31.44	759.3 ± 43.84
MPV (fL)	7.03 ± 0.67	8.27 ± 0.18	7.97 ± 0.17	7.67 ± 0.09	8.67 ± 0.09	8.60 ± 0.40	8.20 ± 0.23

Data are expressed as Mean ± SEM; **significant *p*-value when control is compared with male and female combined *significant *p*-value when control is compared with treatment groups (male and females separated). n (number of rats) = 6/group for treated groups while 5/group for control for the combined analysis. *n* = 3/group for the separate group analysis

### Biochemical indices

Biochemical analysis showed no significant (*p* > 0.05) variations in urea, Aspartate transaminase (AST), Alanine transaminase (ALT), sodium, and potassium values among the different treatment groups compared to the control except creatinine and chloride levels (Table 4). Creatinine levels significantly increased in female rats treated with graded doses of *A. vera* green rind extract. Also, the mean chloride ion significantly increased in rats treated with *A. vera* whole leaf extract while significantly decreased in females treated with *A. vera* green rind extract. Potassium values in female rats treated with 400 mg/kg of whole leaf and rind extracts significantly decreased compared to their respective controls.

**Table 4:** Biochemical profile of rats treated with *A. vera* for 28 days

Biochemical parameters	Control group	***A. vera*** whole leaf extract dose levels in mg/kg			***A. vera*** green rind extract dose levels in mg/kg		
		200	400	800	200	400	800
Combined male and female							
CREAT (mg/dl)	0.57 ± 0.03	0.48 ± 0.04	0.42 ± 0.06	0.49 ± 0.09	0.86 ± 0.07**	0.85 ± 0.06**	0.83 ± 0.06**
Urea (mg/dl)	44.90 ± 3.16	48.42 ± 2.70	48.38 ± 2.80	44.32 ± 3.20	50.15 ± 4.63	45.40 ± 3.22	38.57 ± 3.58
ALT (U/L)	69.40 ± 8.19	65.83 ± 2.36	60.17 ± 5.08	70.50 ± 4.72	104.00 ± 12.86	87.50 ± 13.02	102.50 ± 11.18
AST (U/L)	147.40 ± 13.08	138.50 ± 10.82	139.70 ± 9.53	125.70 ± 2.86	136.00 ± 4.41	128.00 ± 10.13	155.80 ± 15.03
Tot Chol (mg/dl)	50.40 ± 4.08	54.17 ± 5.65	41.67 ± 3.45	52.50 ± 5.93	107.50 ± 19.67	121.70 ± 14.62**	143.0 ± 12.40**
Na + (mmol/l)	147.80 ± 1.04	147.70 ± 0.45	147.20 ± 0.74	147.60 ± 0.61	146.60 ± 0.32	145.90 ± 0.55	145.60 ± 0.39
K+ (mmol/l)	6.98 ± 0.13	6.06 ± 0.16	6.10 ± 0.48	5.97 ± 0.13	6.06 ± 0.19	6.12 ± 0.37	6.37 ± 0.36
Cl- (mmol/l)	111.40 ± 2.81	154.60 ± 4.29**	145.30 ± 5.58**	148.20 ± 8.18**	103.80 ± 0.61**	104.80 ± 0.39**	105.30 ± 0.73**
Females							
CREAT (mg/dL)	0.58 ± 0.02	0.43 ± 0.03	0.41 ± 0.05	0.67 ± 0.06	0.95 ± 0.09*	0.93 ± 0.06*	0.90 ± 0.02*
Urea (mg/dL)	49.05 ± 3.55	50.60 ± 5.36	48.07 ± 2.35	41.73 ± 1.61	57.90 ± 6.21	48.23 ± 6.15	43.87 ± 1.72
ALT (U/L)	53.50 ± 10.50	65.33 ± 2.40	59.00 ± 4.93	71.67 ± 6.64	85.33 **±** 9.96	59.33 ± 5.49	101.00 ± 23.18
AST (U/L)	155.00 ± 9.00	148.7 ± 15.94	140.70 ± 3.84	129.30 ± 3.18	134.00 ± 7.21	121.30 ± 20.46	172.30 ± 7.22
Tot Chol (mg/dL)	49.05 ± 1.50	64.33 ± 4.49	46.67 ± 5.81	60.67 ± 4.67	145.00 ± 5.69*	113.70 ± 30.56	168.70 ± 9.40*
Na + (mmol/L)	147.60 ± 0.95	147.30 ± 0.47	147.70 ± 1.15	146.60 ± 0.84	146.20 ± 0.23	145.90 ± 0.89	145.20 ± 0.73
K+ (mmol/L)	6.71 ± 0.18	5.87 ± 0.15	5.26 ± 0.38*	5.81 ± 0.20	5.74 ± 0.26	5.62 ± 0.05*	5.98 ± 0.20
Cl-(mmol/L)	111.11 ± 0.55	147.30 ± 3.00*	139.70 ± 10.82*	133.30 ± 9.91*	102.80 ± 0.42*	104.30 ± 0.69*	104.90 ± 0.55*
Males							
CREAT (mg/dL)	0.56 ± 0.06	0.53 ± 0.08	0.42 ± 0.12	0.30 ± 0.01	0.76 ± 0.10	0.76 ± 0.08	0.75 ± 0.10
Urea (mg/dL)	42.13 ± 4.42	46.23 ± 1.69	48.70 ± 5.81	46.90 ± 6.48	42.40 ± 2.95	42.57 ± 2.45	33.27 ± 5.76
ALT (U/L)	80.00 ± 6.81	66.33 ± 4.67	61.33 ± 10.17	69.33 ± 8.11	122.7 ± 19.46	115.70 ± 4.91	104.00 ± 9.24
AST (U/L)	142.30 ± 22.60	128.30 ± 15.10	138.70 ± 20.93	122.00 ± 4.16	138.00 ± 6.43	134.70 ± 7.06	139.30 ± 28.39
Tot Chol (mg/dL)	51.00 ± 7.37	44.00 ± 6.03	36.67 ± 0.88	44.33 ± 9.33	70.00 ± 22.27	129.70 ± 8.41*	117.30 ± 4.67*
Na + (mmol/L)	148.00 ± 1.80	148.10 ± 0.78	146.70 ± 1.07	148.60 ± 0.41	147.00 ± 0.56	145.80 ± 0.85	146.00 ± 0.28
K+ (mmol/L)	7.17 ± 0.05	6.25 ± 0.26	6.93 ± 0.56	6.13 ± 0.15	6.38 ± 0.09	6.62 ± 0.66	6.76 ± 0.67
Cl-(mmol/L)	111.60 ± 5.11	161.90 ± 5.51*	151.00 ± 2.56*	163.20 ± 3.63*	104.80 ± 0.84	105.30 ± 0.17	105.80 ± 1.47

Data are expressed as Mean ± SEM; **significant *p*-value when control is compared with treatment groups (male and female combined); *significant *p*-value when control is compared with treatment groups (male and female separated) n (number of rats) =6/group for treated groups while 5/group for control for the combined analysis while *n* = 3/group for the separate group analysis

### Histopathological analysis

**Fig. 6:**
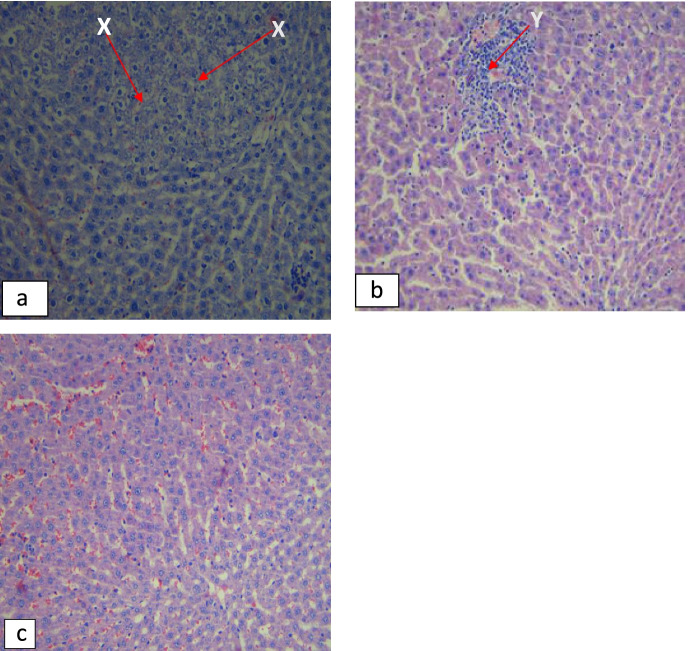
Photomicrographs of the liver of the rats in subacute toxicity. Arrow X shows the necrotic liver tissue while Arrow Y shows inflamed regions of the portal triad

**Fig. 7:**
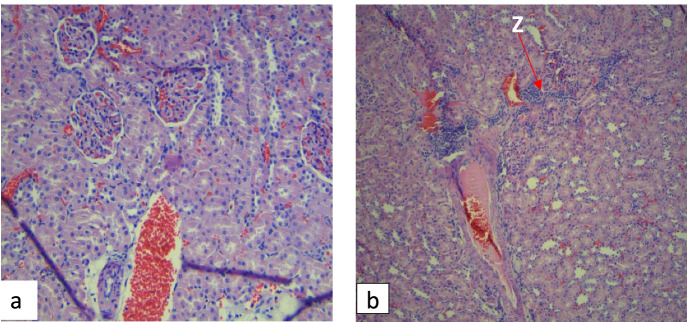
Photomicrographs of the kidney of the rats in subacute toxicity. Arrow Z shows inflamed regions of the renal interstitium

Histopathological examination of the female and male rats’ liver tissue in the control group revealed moderate necrosis (Fig. 6a) and mild inflammation of the portal triad (Fig. 6b). The kidney morphology for all the rats in the control group remained intact (Fig. 7a). The liver tissue of the rats treated with *A. vera* whole leaf and green rind extracts showed moderate necrosis and mild inflammation of the portal triad in male and female rats. The photomicrographs of the kidneys of rats treated with graded doses of *A. vera* green rind extract showed mild necrosis. However, there was mild inflammation of the renal interstitium in some of the rats treated with 200 and 800 mg/kg of *A. vera* whole leaf extract (Fig. 7b).

## Discussion

Acute toxicity testing which involves the estimation of LD_50_ is critical in carrying out toxicological investigations on chemicals, including plant extracts. In the present study, acute oral administration of single doses of the whole leaf and green rind extracts up to 5000 mg/kg neither caused death nor changed the behavior of the animals. Therefore, the LD_50_ for both whole leaf and green rind extracts was above 5000 mg/kg. This value puts the whole leaf and green rind extracts of *A. vera* under GHS Category 5 [32], implying that the extracts are of relatively low acute toxicity. Similarly, the sub-acute oral administration of the whole leaf and green rind extracts caused no mortality and no behavioral changes in the animals throughout the 28-day study period.

There was no significant variation in mean body weight among treatment groups compared with the control. In addition, no significant difference in the mean relative weights of the liver, kidney, brain, ovary, and testes of the rats compared with the control. However, the rats that received 200 mg/kg of *A. vera* green rind extract had significantly decreased relative spleen weight. Alteration in organ weight is usually an indication of the potential toxicity of the substance. The reduction in relative spleen weight could mean that the extract had deleterious effect on it. However more studies need to be conducted on the effect of *A. vera* on the spleen.

Phytochemical investigations revealed tannins, saponins, alkaloids, flavonoids, anthraquinones, and amino acids in the leaves and green rind extracts of *A. vera*. These bioactive constituents are responsible for the *Aloe* species’ different pharmacological activities and some toxicity. The phytochemicals present are in agreement with other studies [19, 20].

The HPLC fingerprint identifies and monitors the quality of different preparations containing *A. vera*. The chromatogram depicts the retention time of each of the peaks. Retention time is usually characteristic for a specific compound in a given preparation. In the present study, fingerprints for whole leaf and green rind extracts indicated that peaks 17 and 15, respectively have the same retention time. Similarly, a well resolved shared peak number 30 was eluted at the same retention time. This indicates that those peaks represent compounds present in both extracts of *A. vera*. Therefore, these two peaks could be regarded as diagnostic peaks for *A. vera* preparations if all other parameters are similar.

The hematopoietic system is more sensitive to the effect of toxic compounds [33]. Therefore, assessing hematological parameters is essential in establishing the effect of plant extracts on the animal’s blood system [34]. In this study, most of the investigated hematological parameters did not show significant variations compared to the control except for the mean values of eosinophils, Hb, HCT, and MPV, which significantly increased while MCH and MCHC levels significantly decreased. A high eosinophil count usually indicates allergic reaction, infections, cancer, and autoimmune diseases [35]. Probably the whole leaf extract of *A. vera* has an effect on the eosinophil levels but this needs to be investigated further.

On the other hand, high levels of Hb and HCT are usually associated with cardiovascular diseases [36]. For the present study the high Hb and HCT levels were not considered to be of toxicity since they were still in the normal range of (13.7–16.8) g/dL and (37.9–49.9) % respectively for female Wistar rats aged 8–16 weeks [37]. Contrary to our findings, Chen et al. found no significant changes in Hb and HCT levels in rats that received *A. vera* whole leaf powder at 400, 1200, and 2000 mg/kg in rats [22].

Mean Corpuscular Hemoglobin (MCH) significantly decreased in male rats treated with 200 and 800 mg/kg of *A. vera* whole leaf and 200 mg/kg of *A. vera* green rind extract. Mean Corpuscular Hemoglobin Concentration levels also significantly decreased in female rats dosed with 200 and 400 mg/kg of *A. vera* green rind extract. Low MCH and MCHC indicate that the extracts make the red blood cells smaller (microcytic), reducing their hemoglobin carrying capacity. Besides, reduced MCH and MCHC levels indicate diminished oxygenation of the tissues [35]. These results differ from a study by Chen et al. where no significant variations were seen in the MCH and MCHC levels when *Aloe vera* whole leaf freeze-dried powder (AWFP) was administered to rats [22]. The discrepancies could be due to the differences in the preparation method. AWFP is a product manufactured by Evergreen Inc. (Kunming, Yunnan Province, China) by freeze-drying technology to treat constipation. It contains mainly aloin and polysaccharides as the active ingredients. The other actives that would have caused toxic effects may have been excluded, leading to the discrepancies in results observed. The MCHC levels decreased below the reference range of (33.2–37.9) g/dL for female Wistar rats aged 8–16 weeks [37] hence of toxicological significance. *A. vera* green rind extract might lower the mean corpuscular hemoglobin concentration, but further studies need to be carried out.

Often, the mean platelet volume (MPV) is linked to platelet size. Thus, it can be used to draw conclusions about platelet count. Our study showed a dose-dependent increase in mean MPV values in female rats treated with 200 and 400 mg/kg of *A. vera* whole leaf and green rind extracts. An elevation in the MPV levels is usually associated with atherothrombotic disorders like myocardial ischemia, cerebrovascular conditions, and atherosclerosis [38]. The increased levels of MPV were not considered as toxicity because they were still in the normal range of (6.2–9.8) fL for Wistar female rats aged 8–16 weeks [37]. However, these results disagree with Archibong et al.*,* where MPV levels significantly decreased in rats treated with a high salt diet combined with 600 mg/kg of *A. vera* gel extract [39].

Assessment of biochemical parameters is critical in assessing organ function, most especially kidney and liver. Non-significant differences were seen in most biochemical parameters (urea, AST, ALT, and sodium values) compared to the control for both males and females except creatinine, chloride, potassium, and total cholesterol values. In male rats, Cl^−^ levels increased compared to the control, and in female rats, creatinine levels increased while K^+^ and Cl^−^ levels decreased compared with the control group. Creatinine is usually formed from creatine’s nonenzymatic breakdown (a protein usually made in the liver) in the muscle, the rate depending on the muscle mass [40]. This creatinine, in turn, is cleared from the body via urine by the kidney. If renal clearance is impaired, serum creatinine levels increase above normal. This makes creatinine an essential biomarker of kidney function. In this study, creatinine levels significantly increased in the female rats treated with graded doses of *A. vera* green rind extract. Elevation in serum creatinine levels is usually associated with kidney injury [41]. This is evidenced by the histopathological examination of the kidney tissue that showed mild necrosis. The increased in the mean creatinine levels in female rats treated with graded doses of *A. vera* green rind extract were considered toxicological significant since the values were out of the reference range of (0.2–0.6) mg/dL for female Wistar rats aged 8–16 weeks [37]. These results are supported by a review done by Boudreau and Beland [42], which stated that *Aloe* ingestion is associated with kidney dysfunction. Therefore *A. vera* green rind extract may be associated with kidney toxicity.

Electrolytes play an essential role in maintaining electrical neutrality, generating and conducting electrical impulses inside the cell and other cells. The kidneys keep working hard to maintain the electrolyte concentration fairly constant despite any changes in the body system [43]. In this study, chloride ion values significantly increased in male rats dosed with graded doses of *A. vera* whole leaf extract but decreased in female rats treated with graded doses of *A. vera* green rind extract. High serum chloride values above normal are usually associated with kidney injury [44], while low serum chloride values are usually attributed to its excess elimination via urine [45]. These results agree with the study by Saka et al. that suggests oral ingestion of *A. vera* is associated with electrolyte imbalance [46]. Furthermore, the increased chloride ion levels were of toxicological significance since the values were out of the reference range of (100–107) mmol/L for female Wistar rats aged 8–16 weeks [37]. Therefore, *A. vera* whole leaf extract may be associated with electrolyte imbalance.

There was an increase in ALT levels for graded doses of *A. vera* green rind extracts though not statistically significant. Alanine aminotransferase is more specific to the liver and is released from the liver cells into the bloodstream when they are damaged. Therefore, high levels of ALT may indicate a liver problem. Our findings showed that the mean ALT levels in treated and control female rats were out of the reference range of (16–48) U/L [37]. This observation concurs with the histopathological findings of the liver tissues. However, such changes were considered environment-related since similar effects occurred in the control groups.

Histopathological examination on the liver showed moderate necrosis and mild inflammation of the portal triad in the treated animals, including the control animals. However, since similar structural changes were also observed in the control groups, it was considered that it might have been due to environmental factors and not related to the extracts. Histopathological examination on the kidney tissue revealed mild inflammation of the renal interstitium in females treated with 200 and 800 mg/kg of *A. vera* whole leaf extract. The toxic effect on the organs may be due to some of the phytochemicals present in the extracts. Phytochemical investigations in this study revealed an adequate amount of tannins. A study conducted by Yamasaki et al. suggests that the intake of large amounts of tannins may cause liver and kidney damage [47]. This study’s results agree with a study conducted by Bala et al. where *A. vera* gel extract caused structural changes in the animals’ kidney tissues at higher doses [20], suggesting that *A. vera* whole leaf extract at high doses may be associated with kidney toxicity.

The differences in the toxicity patterns of the extracts between male and female rats may be due to higher glomerular filtration rate in males, sex-related differences in drug-metabolizing enzymes and or transport proteins, the higher fat percentage in males than females, lower body weight, and organ size in females [48]. This study could not ascertain whether the decrease in relative spleen weight was due to the extract. When using animal models for toxicity studies, some subjective effects like headaches, nausea, dizziness, and mental disturbances cannot be revealed. Toxic effects usually vary from one species to another, but the toxicity data generated from animal studies can be easily extrapolated to humans.

## Conclusion

The whole leaf and green rind extracts of *A. vera* can be considered practically non-toxic for a single dose or short duration administration with an estimated LD_50_ above 5000 mg/kg. However, the sub-acute oral toxicity study findings indicated that *A. vera* might cause kidney toxicity when used for long periods at high doses. Thus, individuals who use *A. vera* drinks daily need to be cautious and should undergo regular kidney function tests for monitoring purposes.

## Supplementary Information

**Additional file 1.** The ARRIVE guidelines 2.0: author checklist.

## Data Availability

The datasets used and/or analyzed during the current study are available from the corresponding author on reasonable request.

## References

[1] Yuan H, Ma Q, Ye L, Piao G (2016). The traditional medicine and modern medicine from natural products. Molecules..

[2] Chikezie PC, Ojiako OA (2015). Herbal medicine: yesterday, today, and tomorrow. Alter Integr Med.

[3] Olaniyan JM, Muhammad HL, Makun HA, Busari MB, Abdullah AS (2016). Acute and sub-acute toxicity studies of aqueous and methanol extracts of *Nelsonia campestris* in rats. J Acute Dis.

[4] Mensah ML, Komlaga G, Forkuo AD, Firempong C, Anning AK, Dickson RA (2019). Toxicity and safety implications of herbal medicines used in Africa. Herbal Med.

[5] Welz AN, Emberger-Klein A, Menrad K (2018). Why people use herbal medicine: insights from a focus-group study in Germany. BMC Complement Altern Med.

[6] Ghosh D, Mondal S, Ramakrishna K (2019). Acute and sub-acute (30-day) toxicity studies of *Aegialitis rotundifolia* Roxb leaves extract in Wistar rats: safety assessment of a rare mangrove traditionally utilized as pain antidote. Clin Phytosci.

[7] Adams K, Eliot T, Gerald A (2014). Extent of use of *Aloe vera* locally extracted products for Management of Ailments in communities of Kitagata sub-county in Sheema District, Western Uganda. Intern J Sci Basic Appl Res.

[8] Kumar S, Yadav JP (2014). Ethnobotanical and pharmacological properties of *Aloe vera*: a review. J Med Plant Res.

[9] El Sayed AM, Ezzat SM, El Naggar MM, El Hawary SS (2016). In vivo diabetic wound healing effect and HPLC–DAD–ESI–MS/MS profiling of the methanol extracts of eight *Aloe* species. Rev Bras.

[10] Dharajiya D, Pagi N, Jasani H, Patel P (2017). Antimicrobial activity and phytochemical screening of *Aloe vera (Aloe barbadensis* miller). Int J Curr Microbiol App Sci.

[11] López A, De Tangil MS, Vega-Orellana O, Ramírez AS, Rico M (2013). Phenolic constituents, antioxidant and preliminary antimycoplasmic activities of whole leaf skin and flowers of *Aloe vera* (L.) Burm. f. (syn. A. barbadensis mill.) from the Canary Islands (Spain). Molecules.

[12] Debnath T, Ghosh M, Lee YM, Nath NC, Lee KG, Lim BO (2018). Identification of phenolic constituents and antioxidant activity of *Aloe barbadensis* flower extracts. Food Agric Immunol.

[13] Gumisiriza H, Birungi G, Olet EA, Sesaazi CD (2019). Medicinal plant species used by local communities around queen elizabeth national park, maramagambo central forest reserve, and ihimbo central forest reserve, southwestern Uganda. J Ethnopharmacol.

[14] Tugume P, Kakudidi EK, Buyinza M, Namaalwa J, Kamatenesi M, Mucunguzi P, Kalema J (2016). Ethnobotanical survey of medicinal plant species used by communities around Mabira central Forest reserve, Uganda. J Ethnobiol Ethnomed.

[15] Anywar G, Kakudidi E, Byamukama R, Mukonzo J, Schubert A, Oryem-Origa H (2020). Indigenous traditional knowledge of medicinal plants used by herbalists in treating opportunistic infections among people living with HIV/AIDS in Uganda. J Ethnopharmacol.

[16] Jadid N, Kurniawan E, Himayani CE, Prasetyowati I, Purwani KI, Muslihatin W, Hidayati D, Tjahjaningrum IT (2020). An ethnobotanical study of medicinal plants used by the Tengger tribe in Ngadisari village, Indonesia. PLoS One.

[17] Hossain MS, Mamun-Or-Rashid AN, Towfique NM, Sen MK (2013). A review on ethnopharmacological potential of *Aloe vera* L. J Complement Med Res.

[18] Sharma P, Kharkwal AC, Kharkwal H, Abdin MZ, Varma A (2014). A review on pharmacological properties of *Aloe vera*. Int J Pharm Sci Rev Res.

[19] Surjushe A, Vasani R, Saple DG (2008). *Aloe vera*: a short review. Indian J Dermatol.

[20] Bala S, Chugh NA, Bansal SC, Garg ML, Koul A (2017). Safety evaluation of *Aloe vera* pulp aqueous extract based on histoarchitectural and biochemical alterations in mice. Indian J Exp Biol.

[21] Nghonjuyi NW, Tiambo CK, Taïwe GS, Toukala JP, Lisita F, Juliano RS, Kimbi HK (2016). Acute and sub-chronic toxicity studies of three plants used in Cameroonian ethnoveterinary medicine: *Aloe vera* (L.) Burm. f. (Xanthorrhoeaceae) leaves, *Carica papaya* L. (Caricaceae) seeds or leaves, and *Mimosa pudica* L. (Fabaceae) leaves in Kabir chicks. J Ethnopharmacol.

[22] Chen T, Wang L, Hu C (2017). Treatment-related changes after short-term exposure of S.D. rats to *Aloe vera* whole-whole leaf freeze-dried powder. Intern. J Exp Pathol.

[23] Erhabor JO, Idu M (2017). Aphrodisiac potentials of the ethanol extract of *Aloe barbadensis* mill. Root in male Wistar rats. BMC Complement Altern Med.

[24] Sodani IJ (2015). Histopathological changes of male mice kidneys treated with fresh *Aloe vera* whole leaf extract. Iraqi J Med Sci.

[25] IARC (2015). *Aloe vera*. Some drugs and herbal products.

[26] OECD Test guideline 425: acute oral toxicity - up-and-down procedure, Guideline for Testing of Chemicals 2001. 10.1787/9789264071049-en.

[27] OECD_407. Test No. 407: repeated dose 28-day Oral toxicity study in rodents. OECD; 2008. 10.1787/9789264070684-en.

[28] Evans WC (2009). Trease and Evans Pharmacognosy.

[29] National Research Council (2011). Guide for care and use of laboratory animals.

[30] Percie du Sert N, Hurst V, Ahluwalia A, Alam S, Avey MT, Baker M, Browne WJ, Clark A, Cuthill IC, Dirnagl U, Emerson M, Hurst V, Ahluwalia A, Alam S, Avey MT (2020). The ARRIVE guidelines 2.0: updated guidelines for reporting animal research. J Cereb Blood Flow Metab.

[31] Wu JY, Chan YC, Guo H, Chen YJ, Liu YX, Yi H, Yu ZL (2020). Twenty-four-week oral dosing toxicities of *Herba Siegesbeckiae* in rats. BMC Complement Med Ther.

[32] Bulgheroni A, Kinsner-Ovaskainen A, Hoffmann S, Hartung T, Prieto P (2009). Estimation of acute oral toxicity using the No observed adverse effect level (NOAEL) from the 28-day repeated dose toxicity studies in rats. Regul Toxicol Pharmacol.

[33] Mukinda JT, Syce JA (2007). Acute and chronic toxicity of the aqueous extract of *Artemisia afra* in rodents. J Ethnopharmacol.

[34] Yakubu MT, Akanji MA, Oladiji AT (2007). Hematological evaluation in male albino rats following chronic administration of aqueous extract of *Fadogia agrestis* stem. Pharmacogn Mag.

[35] Arika WM, Nyamai DW, Musila MN, Ngugi MP, Njagi EN (2016). Hematological markers of in vivo toxicity. J Hematol Thromboembol Dis.

[36] Kishimoto S, Maruhashi T, Kajikawa M, Matsui S, Hashimoto H, Takaeko Y, Harada T, Yamaji T, Han Y, Kihara Y, Chayama K (2020). Hematocrit, hemoglobin and red blood cells are associated with vascular function and vascular structure in men. Sci Rep.

[37] Giknis ML, Clifford CB (2008). Clinical laboratory parameters for Crl: Wi (Han) rats.

[38] Şenel E, Acar B, Demir E (2017). Mean platelet volume: A reliable marker of inflammation in recurrent apthous stomatitis and behcet disease?. Indian Dermatol Online J.

[39] Archibong AN, Nku CO, Udefa AL, Leilei SA (2018). *Aloe vera* gel reverses hemostatic derangement in rats following salt loading. World J Pharm Res.

[40] Zuo Y, Wang C, Zhou J, Sachdeva A, Ruelos VC (2008). Simultaneous determination of creatinine and uric acid in human urine by high-performance liquid chromatography. Anal Sci.

[41] Oloyede OB, Sunmonu TO (2009). Potassium bromate content of selected bread samples in Ilorin, Central Nigeria and its effect on some enzymes of rat liver and kidney. Food Chem Toxicol.

[42] Boudreau MD, Beland FA (2006). An evaluation of the biological and toxicological properties of *Aloe barbadensis* (miller), *Aloe vera*. J Environ Sci Health Part C.

[43] Shrimanker I, Bhattarai S. Electrolytes. InStatPearls 2020. StatPearls Publishing. 31082167

[44] Zhang Z, Xu X, Fan H, Li D, Deng H (2013). Higher serum chloride concentrations are associated with acute kidney injury in unselected critically ill patients. BMC Nephrol.

[45] Berend K, van Hulsteijn LH, Gans RO (2012). Chloride: the queen of electrolytes?. Eur J Intern Med.

[46] Saka WA, Akhigbe RE, Popoola OT, Oyekunle OS (2012). Changes in serum electrolytes, urea, and creatinine in *Aloe vera*-treated rats. J Young Pharm.

[47] Yamasaki T, Sato M, Mori T, Mohamed AS, Fujii K, Tsukioka J (2002). Toxicity of tannins towards the free-living nematode *Caenorhabditis elegans* and the brine shrimp (*Artemia salina)*. J Nat Toxins.

[48] Sakuma T, Kawasaki Y, Jarukamjorn K, Nemoto N (2009). Sex differences of drug-metabolizing enzyme: female predominant expression of human and mouse cytochrome P450 3A isoforms. J Health Sci.

